# Atomic site engineering in garnet phosphors taming thermal quenching for high-power visible and NIR luminescence

**DOI:** 10.3389/fchem.2026.1986069

**Published:** 2026-10-30

**Authors:** Jiajun Feng, Peixuan Chen, Yuyuan Chen, Keyi Fang, Shiting Chen, Xiaofeng Li, Yanjing Wu, Liyuan Huang, Xiang Li

**Affiliations:** School of Environmental and Chemical Engineering, New Energy and New Materials Research Center, Zhaoqing University, Zhaoqing, China

**Keywords:** crystal field engineering, garnet phosphors, luminescence stability, near-infrared phosphors, site engineering, thermal quenching

## Abstract

Rare-earth (RE) and transition metal-doped garnet phosphors are the dominant commercial luminescent materials for high-power white light-emitting diodes (wLEDs), and emerging near-infrared (NIR) phosphors for spectroscopy, bioimaging and plant lighting. A long-standing bottleneck for high-power operation is thermal quenching: commercial YAG:Ce^3+^ typically loses >20% of its room-temperature emission intensity at 150 °C, the standard operating temperature of 1 A-driven pc-LEDs, leading to efficiency roll-off, color shift and shortened device lifetime. Unlike passive strategies such as surface coating or glass encapsulation, site engineering modulates the local coordination environment of luminescent centers at the atomic scale, providing an intrinsic route to improve thermal stability without sacrificing quantum efficiency. This mini review summarizes the structure-property relationship between crystallographic site properties and thermal quenching behavior, critically evaluates three mainstream site engineering approaches (isovalent substitution, aliovalent charge-compensated co-doping, multi-site occupancy design) with representative material systems covering both RE (Ce^3+^, Eu^3+^) and transition metal (Cr^3+^, Mn^2+^/Mn^4+^) activators, and highlights unresolved research gaps.

## 1 Introduction

Garnet phosphors with the general formula A_3_B_2_C_3_O_12_ (space group $Ia\bar{3}d$ ) have been the workhorse of solid-state lighting for nearly 3 decades, starting with the commercialization of YAG:Ce^3+^ for wLEDs in the 1990s (Xia and Meijerink, 2017; Ma et al., 2025; Nasir et al., 2026; Tikale et al., 2026; Zhao et al., 2026a). The rigid three-dimensional framework, excellent chemical stability, and flexible cation occupancy make garnets uniquely suitable for activator doping: Ce^3+^, Cr^3+^, Mn^2+^/Mn^4+^ and other lanthanide/transition metal activators can be incorporated into different crystallographic sites, producing emission covering the entire visible to NIR range (George et al., 2013; Fan et al., 2026). In recent years, Cr^3+^-doped garnet NIR phosphors have attracted particular attention for non-destructive food testing, *in vivo* bioimaging, and plant growth lighting, with multiple materials already moving to commercialization (Rajendran et al., 2022). The concurrent shift toward laser-diode excitation and integrated short-wave infrared sources demands emitters that remain stable under far higher flux densities, and Ni^2+^-doped transparent ceramics now extend such broadband site-engineering concepts into the short-wave infrared window (Feng et al., 2026a; Zhao et al., 2026b). Beyond Cr^3+^-based NIR-I/II emission, lanthanide co-doped systems are now extending the response into the NIR-III window (Feng et al., 2026).

For high-power device applications, however, thermal quenching remains the most critical performance limitation. At driving currents above 1 A, the junction temperature of pc-LEDs can easily reach 200 °C–250 °C, where non-radiative transition rates increase exponentially, leading to severe emission loss (Lin et al., 2019; Liang et al., 2023). For high-temperature luminescence thermometry and scintillation applications, thermal stability is even more critical to ensure measurement accuracy and radiation hardness (Yadav et al., 2015; Wang et al., 2023; Bindhu et al., 2025; Feng et al., 2026b; Liang et al., 2026). A range of strategies have been developed to mitigate thermal quenching, including surface coating, glass ceramic encapsulation, defect engineering and bandgap engineering (Fasoli et al., 2011; Vrubel et al., 2017; Kuk et al., 2019). Among these, site engineering is particularly attractive because it modifies the intrinsic properties of the phosphor lattice rather than adding passive external layers, making it compatible with conventional ceramic synthesis and large-scale manufacturing (Lang et al., 2022; Feng et al., 2025).

A number of comprehensive reviews have summarized garnet phosphor development in recent years, covering composition design, spectral tuning, and device applications (Chen and Huang, 2026). However, existing reviews either provide a broad overview of all phosphor families, focus exclusively on spectral tuning for specific activators, or discuss thermal stability as a secondary performance metric rather than a core design target. To date, no focused mini review has systematically addressed how atomic-scale site engineering can be used to regulate thermal quenching behavior in garnet phosphors, or compared the advantages and limitations of different site modification strategies. This work fills this gap by providing a concise, critical overview of site-engineered thermal stability enhancement in garnet phosphors.

## 2 Mechanistic basis of site-dependent thermal quenching

### 2.1 Core thermal quenching mechanisms in garnets

Thermal quenching in activator-doped garnets arises from four primary physical mechanisms, whose relative contribution varies with activator type, doping concentration, and lattice structure (Blasse et al., 1995; Hu et al., 2026). The most widely cited mechanism is thermal crossover: at elevated temperatures, excited electrons gain sufficient thermal energy to overcome the activation barrier Δ*E* between the excited state and ground state parabolas in the configurational coordinate diagram, leading to non-radiative relaxation (Feng et al., 2024a; Song et al., 2025).

The second mechanism is thermal ionization, which is physically distinct from crossover: an excited electron is promoted thermally from the emitting level into the host conduction band, migrates through the lattice, and is ultimately captured non-radiatively by defects or traps, instead of returning through the crossing of the excited- and ground-state parabolas (Romero et al., 2026). Because its rate is governed by the energy separation between the emitting level and the conduction-band minimum rather than by the crossover barrier *ΔE*, thermal ionization responds differently to composition and cannot be distinguished from crossover by intensity-versus-temperature curves alone; temperature-dependent photoconductivity, thermoluminescence or excited-state lifetime measurements are required (Kieu et al., 2026).

Which channel dominates is strongly activator- and host-dependent, and the community has moved toward a differentiated rather than a universal picture. For Ce^3+^-doped aluminate garnets, early work attributed quenching entirely to configurational crossover, whereas photoconductivity, thermoluminescence and first-principles studies now indicate that thermal ionization from the lowest 5*d* level to the conduction band is the dominant pathway in YAG:Ce^3+^, because the 5*d*_1_ level lies comparatively close to the conduction-band minimum; lowering the conduction band through Ga substitution further facilitates ionization and can even lead to direct, athermal ionization in gallium-rich garnets (Lin et al., 2019; Fasoli et al., 2011; Vrubel et al., 2017). This ionization-centered interpretation is increasingly, though not universally, accepted: measurements that detected no ionization-related delayed recombination have instead been taken as evidence for crossover, and the crossover-to-ionization balance shifts continuously with bandgap and 5*d*-level position. The debate for Ce^3+^ garnets is therefore converging rather than fully closed, and it should not be conflated with the separate, concentration-dependent role of defect migration noted above. For Cr^3+^-doped garnets the 3*d* emitting levels lie deep inside a wide bandgap and thermal ionization is energetically costly; quenching is instead governed by ^4^T_2_–^4^A_2_ configurational crossover, together with thermally driven population redistribution between the spin-forbidden ^2^E and spin-allowed ^4^T_2_ levels, which in rigid hosts can yield near-zero or anti-thermal quenching (Zou et al., 2024). For *f*–*f* activators such as Eu^3+^, the large protective energy gap suppresses both crossover and ionization, leaving multiphonon relaxation and energy migration to defects as the rate-limiting steps (Sao et al., 2025; Chen et al., 2026). A practical consequence is that the dominant mechanism cannot be assumed *a priori* and must be identified for each activator–host combination before a site engineering strategy is chosen.

The third mechanism is phonon-assisted non-radiative transition: increased lattice vibration at high temperatures enhances electron-phonon coupling, allowing excited electrons to relax via multi-phonon emission rather than radiative photon emission (Wei et al., 2025). This mechanism is particularly sensitive to lattice rigidity, as softer lattices with lower phonon energy show stronger non-radiative coupling. The fourth mechanism is energy-migration-coupled quenching: at high temperatures, excitation energy can migrate over longer distances through the activator sublattice, eventually being trapped by defects or impurity quenching centers (Wang et al., 2020). This mechanism explains the common observation that thermal stability often decreases sharply above a critical doping concentration, as shorter inter-activator distances enable faster energy migration. Notably, multiple recent studies have reported abnormal anti-thermal quenching behavior in modified garnets, where emission intensity increases with temperature up to 200 °C–300 °C (Balhara et al., 2024; Zhu et al., 2024). This effect typically arises either from thermally activated electron release from shallow defect traps, or from temperature-dependent electron redistribution between thermally coupled energy levels of the activator, which can compensate for non-radiative losses and even produce net emission enhancement (Zuo et al., 2022; Zhou et al., 2024; Cheng et al., 2026).

### 2.2 How local site properties control thermal stability

The thermal stability of an activator center is directly determined by its local coordination environment, which can be precisely tuned via site engineering. Three key structural parameters dominate thermal quenching behavior.

First, site symmetry and crystal field splitting set the activation energy for thermal crossover. The magnitude of crystal field splitting Δ depends on polyhedral symmetry and metal-ligand bond lengths: lower site symmetry increases crystal field splitting, reducing the energy barrier for thermal crossover and lowering quenching temperature (Song et al., 2018; Gu et al., 2026). For example, polyhedral symmetry breaking in Cr^3+^-doped garnets has been shown to adjust the energy gap between the ^4^T_2_ and ^2^E thermally coupled levels, enabling electron redistribution at high temperatures and producing anti-thermal quenching behavior (Qian et al., 2025b; Gao et al., 2026).

Second, lattice rigidity and bond strength control electron-phonon coupling strength. Shorter, stronger metal-oxygen bonds increase polyhedral rigidity, reduce phonon energy, and suppress lattice vibration at high temperatures, weakening multi-phonon non-radiative transitions (Song et al., 2024; Zhang et al., 2025b). This is the most straightforward route to high thermal stability: rigid hosts such as Lu_3_Al_5_O_12_ consistently show better thermal stability than softer gallium or germanium garnets, and the most thermally stable garnets reported to date all use highly rigid polyhedral frameworks (Feng et al., 2022; Gao et al., 2026).

Third, site isolation modulates long-range energy migration. When activator ions are spatially separated by non-luminescent cations or structural barriers, the critical energy transfer distance between activators increases, suppressing long-range energy migration to quenching centers (Al-Rasbi and Al Riyami, 2026; Liu et al., 2026b). This effect can simultaneously improve both concentration quenching and thermal quenching thresholds, as both processes rely on energy migration through the activator sublattice.

### 2.3 Crystallographic site flexibility in garnet lattices

The garnet structure contains three distinct cation sites, each with different coordination geometry and cation preference, providing independent degrees of freedom for site engineering (Sifat and Grosvenor, 2018). The 8-coordinated dodecahedral A-site typically hosts large cations (Y^3+^, Lu^3+^, Gd^3+^, Ca^2+^, Sr^2+^) and is the primary occupancy site for Ce^3+^ and Eu^3+^ activators. The 6-coordinated octahedral B-site hosts smaller trivalent/divalent cations (Al^3+^, Ga^3+^, Mg^2+^, Sc^3+^, In^3+^) and is the dominant site for Cr^3+^ and Mn^4+^ activators in NIR and far-red phosphors (Malysa et al., 2018; Xu et al., 2025). The 4-coordinated tetrahedral C-site hosts small tetravalent cations (Si^4+^, Ge^4+^) and does not typically accommodate activators, but plays a critical role in determining overall lattice connectivity and rigidity (Tian et al., 2022).

Wide solid solution ranges are possible at all three sites, allowing cation substitution without breaking the garnet structure. This flexibility is the foundation of site engineering: by selecting appropriate substituents for each site, researchers can independently tune crystal field strength, lattice rigidity, defect chemistry and energy migration paths to optimize thermal stability (Li et al., 2026b). The correlations between crystallographic sites, thermal quenching pathways and corresponding site engineering strategies are schematically illustrated in Figures 1a–c.

**FIGURE 1:**
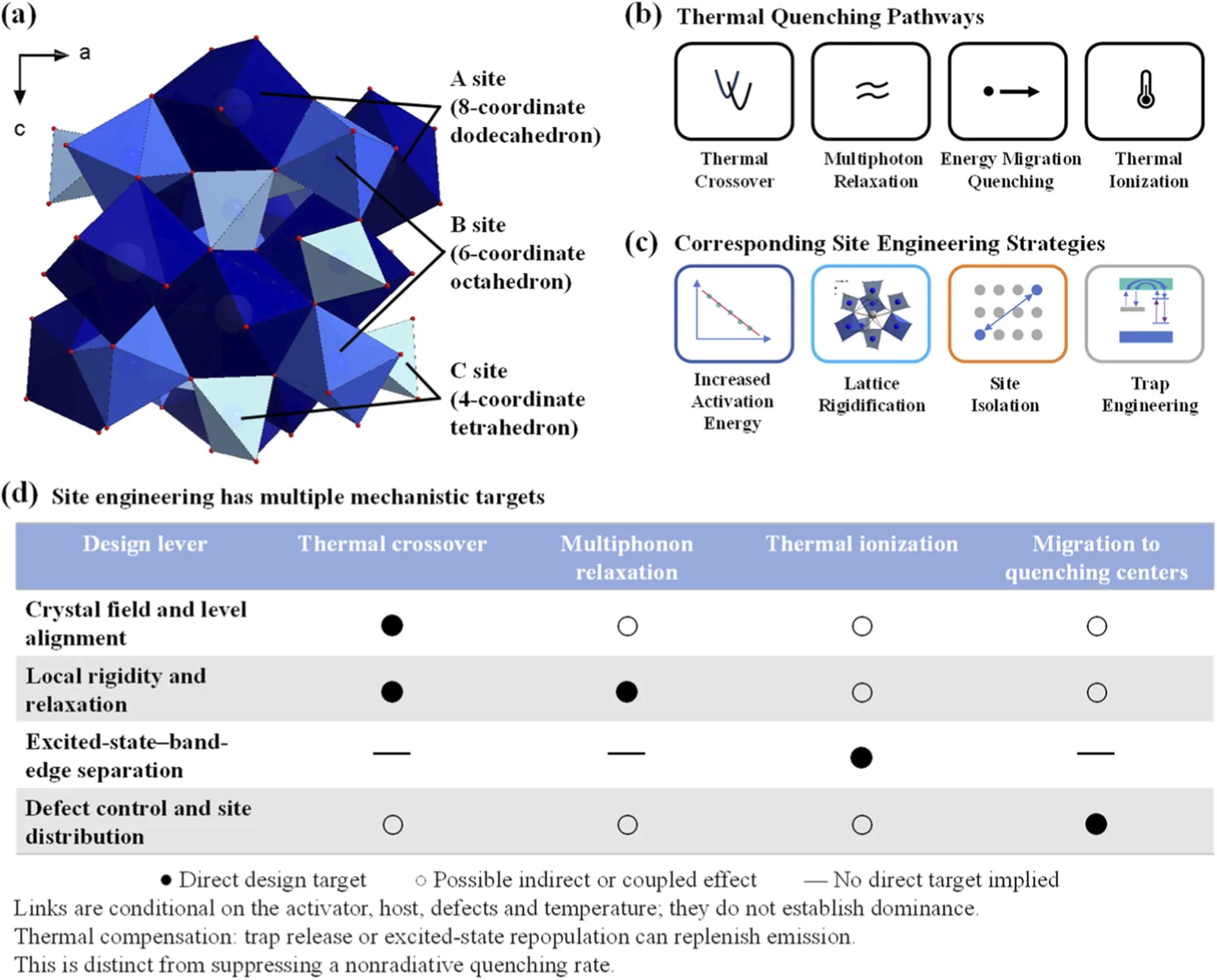
Mechanistic schematic of site engineering for thermal quenching regulation in garnet phosphors. **(a)** Garnet unit cell structure highlighting A (8-coordinate dodecahedral), B (6-coordinate octahedral) and C (4-coordinate tetrahedral) cation sites. **(b)** Four core thermal quenching pathways: thermal crossover, multiphonon relaxation, energy migration quenching, and thermal ionization. **(c)** Four corresponding site engineering strategies: increased activation energy, lattice rigidification, site isolation, and trap engineering. **(d)** Design-lever–mechanism matrix summarizing the many-to-many relationship between site engineering levers and quenching mechanisms: filled circles denote a direct design target, open circles a possible indirect or coupled effect, and dashes the absence of a direct target, showing that each lever primarily addresses one quenching channel while often coupling to several others.

It should be emphasized that the strategy–mechanism correspondence shown in Figure 1c and summarized as the design-lever–mechanism matrix in Figure 1d is a conceptual guide rather than an exclusive one-to-one map, because the actual relationships are many-to-many and a single substitution usually acts on several quenching channels at once. Lattice rigidification, for example, is directed primarily at multiphonon relaxation, but shorter, stiffer bonds also reduce the equilibrium-coordinate offset between parabolas and hence raise the crossover barrier *ΔE*, while widening the bandgap moves the conduction band away from the emitting level and concurrently suppresses thermal ionization. Crystal-field and site-symmetry control likewise alters both the crossover barrier, through level splitting, and the emitting-level-to-conduction-band gap; site isolation, although designed mainly to interrupt energy migration, also lowers the local defect density at which ionized electrons are captured (Zuo et al., 2026). Conversely, one mechanism may be addressed by several strategies: thermal ionization can be suppressed both by rigidification-induced bandgap widening and by charge compensation that removes vacancy-derived traps, whereas trap engineering does not suppress quenching at all but compensates radiative losses through thermally released carriers. Rational design therefore starts from the dominant mechanism and combines complementary strategies, rather than matching a single structural modification to a single quenching pathway.

## 3 Site engineering strategies for improved thermal stability

### 3.1 Isovalent cation substitution

Isovalent substitution, where a host cation is replaced by another ion of the same charge and similar ionic radius, is the most widely used industrial strategy because it introduces minimal charge defects and is compatible with large-scale solid-state synthesis. A-site isovalent substitution is primarily used to tune lattice rigidity (Wu et al., 2026). Replacing larger A-site cations (Y^3+^, Gd^3+^) with smaller Lu^3+^ reduces lattice parameters, shortens metal-oxygen bonds, and increases overall lattice rigidity, reducing non-radiative transition rates (Huang et al., 2021; Shen et al., 2026). For example, partial Lu^3+^ substitution for Y^3+^ in YAG:Ce^3+^ phosphor ceramics enhances thermal quenching resistance. As reported by Ling et al. (2021), at 250 °C, the luminescence intensity of the optimally doped YLuAG:Ce ceramic decreases by only 8.9%, which is lower than the 11.1% intensity attenuation of undoped YAG:Ce ceramic. The main limitation of A-site substitution is that the performance gains are relatively modest: even at optimal substitution levels, the improvement in thermal stability typically ranges from 10% to 20%.

B-site isovalent substitution is particularly effective for Cr^3+^-doped NIR phosphors, as Cr^3+^ occupies the octahedral B-site. Replacing Al^3+^ with larger Ga^3+^ or In^3+^ expands the octahedron, reduces crystal field strength, and broadens NIR emission, but typically reduces thermal stability due to softer lattice vibrations (Mao et al., 2020; Qian et al., 2025a). Co-substitution of multiple B-site cations can be used to balance emission bandwidth and thermal stability: a notable example is [Lu^3+^-Ga^3+^] co-substitution in Lu_2_CaGa_4_SnO_12_:Cr^3+^, where simultaneous A and B site tuning adjusts thermally coupled level gaps to produce anti-thermal quenching, with 142% emission retention at 425 K. Another representative case is the Cr^3+^-activated Lu_2_BaAl_4_SiO_12_ garnet phosphor: through [Ba^2+^-Si^4+^] chemical unit cosubstitution for [Lu^3+^-Al^3+^], it realizes strong crystal-field NIR emission and anti-thermal quenching performance of 126% at 498 K (Zhou et al., 2024; Cheng et al., 2026).

C-site isovalent substitution (Si^4+^/Ge^4+^ exchange) tunes tetrahedral connectivity and overall lattice rigidity. Partial Si^4+^ substitution for Ge^4+^ in germanate garnets increases bond strength and improves thermal stability while maintaining broad NIR emission (Shen et al., 2024). Mg^2+^-Si^4+^ pair substitution for Al^3+^ across B and C sites is also widely used in aluminate garnets to adjust crystal field and improve thermal stability for both Ce^3+^ and Cr^4+^ emission (Meng et al., 2019; Jia et al., 2020a). Pushing coordinated substitution across several sites still further, structural reconstruction combined with crystal-field engineering in Ca_2_GdZrSnGa_3_O_12_:Cr^3+^ delivers broadband NIR emission stable under both laser-diode and LED excitation (Wu and Jin, 2026), and Sn/Si/Ga site regulation in Ca_3_Sn_2_SiGa_2_O_12_:Cr^3+^ extends broadband emission to longer wavelengths while retaining a rigid framework (Li and Jin, 2026).

The key advantage of isovalent substitution is its manufacturing simplicity, but the modulation range is limited by ionic radius mismatch, and excessive substitution leads to phase separation or increased lattice disorder that degrades performance.

### 3.2 Aliovalent charge-compensated co-doping

Aliovalent substitution, where substituent ions have different charge from the host cation, introduces charge imbalance but can produce much larger changes to local structure and defect chemistry than isovalent substitution. When paired with appropriate charge compensation, aliovalent co-doping can achieve dramatic improvements in thermal stability that are not possible with isovalent substitution alone.

The most common design uses charge-compensated ion pairs to maintain overall charge neutrality. Monovalent alkali ions (Li^+^, Na^+^, K^+^) are frequently co-doped with higher-valence substituents to compensate charge imbalance, eliminating oxygen vacancy defects that act as non-radiative quenching centers (Hu et al., 2019; Yu et al., 2026). For example, Na^+^ co-doping in Na_2_CaZr_2_Ge_3_O_12_:Cr^3+^ and NaY_2_Ga_2_InGe_2_O_12_:Cr^3+^ provides charge compensation for Cr^3+^ incorporation at the B-site while introducing local lattice distortion that increases rigidity, producing materials with >90% emission retention at 150 °C and internal quantum efficiencies above 90% (Liao et al., 2024; Xing et al., 2025).

Aliovalent co-doping can also produce a site isolation effect that suppresses energy migration. Local charge and strain fields around aliovalent substituents create potential barriers between activator ions, increasing the critical energy transfer distance and breaking long-range energy migration paths (Feng et al., 2024b). For example [Na^+^-Ge^4+^] co-substitution in YAG:Ce^3+^ creates local structural distortions that isolate Ce^3+^ activators, increasing the quenching concentration from 3 mol% to 7 mol% and improving 200 °C emission retention by 22% (Hu et al., 2019).

The primary limitation of aliovalent co-doping is that it requires precise dopant control: insufficient charge compensation leaves harmful vacancy defects, while excess co-dopant forms impurity phases or introduces new quenching centers. For this reason, aliovalently doped phosphors often show poorer batch-to-batch reproducibility in large-scale synthesis compared to isovalently substituted materials.

### 3.3 Multi-site occupancy design

In many garnet solid solutions, activators can occupy multiple crystallographic sites simultaneously, creating distinct luminescent centers with different crystal field environments and thermal responses. Rational design of multi-site occupancy and inter-site energy transfer provides an additional route to tune thermal quenching behavior.

Controlled multi-site occupancy is achieved by adjusting composition to tune activator distribution across sites. For example, in Ca_3_Sc_2_Si_3_O_12_, Cr^3+^ occupies both Sc^3+^ octahedral sites and Ca^2+^ dodecahedral sites, producing two NIR emission centers with different thermal stability; tuning the Ca/Sc ratio to direct energy transfer to the more stable center improves overall thermal stability by ∼18% (Jia et al., 2020b; Pei et al., 2026). It should be noted that there remains significant disagreement in the literature about exact site occupancy ratios in many multi-site garnets, with different characterization techniques often producing conflicting results, which remains a major barrier to rational multi-site design.

Dual-site cooperative designs have recently produced some of the highest performance broadband NIR phosphors reported. For example, Gd_3_Zn_2_GaGe_3_O_12_:Cr^3+^ uses Cr^3+^ occupancy at both octahedral and dodecahedral sites to produce ultra-broadband emission covering 700–1100 nm, while energy transfer between sites and rigid lattice structure maintains 81% emission at 150 °C (Peng et al., 2026). Similarly, A/C site co-substitution in YAG:Cr^3+^ creates multiple distinct Cr^3+^ centers with tailored energy transfer, producing broad NIR emission with 90% thermal stability at 423 K (Shu et al., 2026). Multi-site design can also produce anti-thermal quenching via thermally activated energy transfer from higher-energy to lower-energy centers at elevated temperatures (Liu et al., 2026a). The main challenge for multi-site design is precise control of site occupancy: small variations in sintering temperature or atmosphere can significantly change activator distribution, leading to variable performance between batches. Figure 2 provides a systematic classification of the three site engineering approaches and Table 1 summarizes a direct comparison of representative materials, the site engineering strategy employed, the dominant quenching mechanism targeted, and the reported thermal retention or quenching temperature.

**FIGURE 2:**
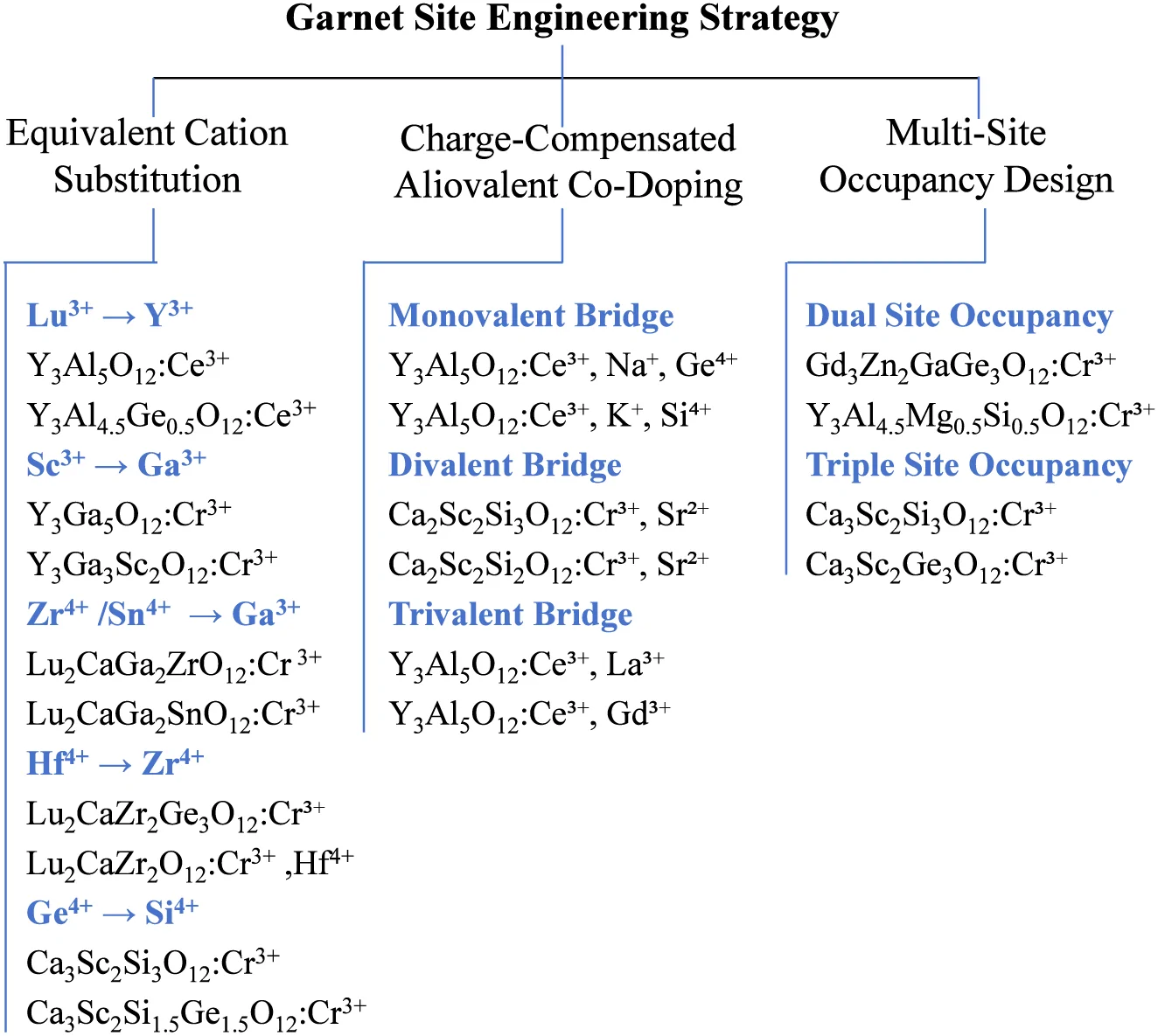
Classification of garnet site engineering strategies. Tree diagram of three mainstream site engineering approaches and their sub-categories, with representative material systems listed for each sub-type.

**TABLE 1:** Direct comparison of representative site-engineered garnet phosphors.

Activator–host garnet	Site engineering strategy	Dominant quenching mechanism addressed	Reported thermal stability metric*
Lu_2_CaGa_4_SnO_12_:Cr^3+^	[Lu^3+^–Ga^3+^] A/B co-substitution; coupled-level tuning	Crossover; population redistribution	142% at 425 K, anti-thermal quenching (Cheng et al., 2026)
Lu_2_BaAl_4_SiO_12_:Cr^3+^	[Ba^2+^–Si^4+^] for [Lu^3+^–Al^3+^] chemical-unit co-substitution	Crossover (strong crystal field)	126% at 498 K (Zhou et al., 2024)
Na_2_CaZr_2_Ge_3_O_12_:Cr^3+^	Na^+^ charge-compensated aliovalent co-doping	Energy migration; defect trapping	>90% at 150 °C; IQE >90% (Liao et al., 2024)
Ca_3_Sc_2_Si_3_O_12_:Cr^3+^	Cr^3+^ occupation at octahedral Sc^3+^ site; weak crystal field modulation via silicate garnet lattice design	Thermal quenching via temperature-dependent lattice vibration; multiphonon relaxation (*f*–*f* activator)	Characterized over 77–573 K; Huang–Rhys parameter *S* = 4; Stokes shift ∼2900 cm^-1^ ( Jia et al., 2020b )
Y_3-*y*_Ca_*y*_Ga_4.95-*y*_Sn_3-*y*_O_12_:Cr^3+^	A-site Ca^2+^ and C-site Sn^4+^ cosubstitution (A&C ligand modulation); octahedral Jahn-Teller distortion engineering	Weak electron-phonon coupling; oxygen vacancy defect-mediated quenching; configurational crossover	91.3% emission intensity retention at 423 K; <5% intensity loss after 30-day operation (Peng et al., 2026)
Gd_3_ScGa_4_O_12_:Cr^3+^	B-site Sc^3+^/Ga^3+^ site reconstruction (1:1 M ratio); crystal field strength reduction; ^4^T_2_ excited-state population optimization	Anti-thermal quenching via excited-state population tuning; weak EPC; structural rigidity effect	198% of room-temperature NIR emission at 543 K (anti-thermal quenching); IQE = 82.75% ( Liu et al., 2024 )
Ca_3_Sn_2_SiGa_2_O_12_:Cr^3+^	Synergistic cation occupation at A/B/C sites; octahedral distortion engineering; pentavalent ion codoping for charge compensation	Configurational crossover; charge-compensation suppressed non-radiative defect pathways	78.03% emission intensity retention at 423 K; 4.3-fold IQE enhancement (Li and Jin, 2026)
Ca_2_GdZrSnGa_3_O_12_:Cr^3+^	Ca–Zr/Sn cooperative substitution; M^5+^ charge compensation; weak-field octahedral environment engineering	Suppressed electron-phonon coupling; configurational crossover; defect passivation by charge compensation	81.58% emission retention at 423 K; activation energy *E*_a_ = 0.25 eV; Huang-Rhys *S* = 0.94 ( Wu and Jin, 2026 )
Y_2_CaGa_4_GeO_12_:Cr^3+^	Ca^2+^ + Ge^4+^ cosubstitution for Y^3+^ + Ga^3+^ in YGG lattice; weak crystal field modulation	EPC-induced thermal quenching; ^4^T_2_–^4^A_2_ configurational crossover	66.4% integrated emission retention at 423 K (vs. 298 K) (Shu et al., 2026)

## 4 Discussion and outlook

Site engineering has enabled dramatic improvements in garnet phosphor thermal stability over the past decade, with state-of-the-art materials now retaining >90% emission at 150 °C (Xie et al., 2025), and some systems showing anti-thermal quenching up to 300 °C (Zong et al., 2023). These advances have enabled higher-power pc-LEDs with improved efficiency and lifetime, but three key challenges remain to be addressed.

First, accurate, quantitative characterization of site occupancy remains elusive. Most studies infer site occupancy indirectly from luminescence spectra or Rietveld refinement of X-ray diffraction data, which cannot provide unambiguous atomic-scale determination of dopant location (Yang et al., 2025). Different characterization methods often produce contradictory occupancy results, making it difficult to establish reliable structure-property relationships. The combination of aberration-corrected STEM, solid-state NMR, and X-ray absorption spectroscopy with first-principles calculation will be required to directly resolve dopant occupancy and local structure at the atomic scale (Chen et al., 2022).

Second, high-temperature *in situ* structural evolution is poorly understood. Almost all structural characterizations are performed at room temperature, but local coordination environments change significantly at operating temperatures due to thermal expansion, polyhedral rotation, and defect migration. These dynamic changes directly determine thermal quenching behavior, but are rarely studied *in situ*. High-temperature *in situ* XRD, Raman and photoluminescence studies will be critical to understand how local structure evolves under real operating conditions (Li and Jin, 2025).

Third, current site design is largely trial-and-error, which is inefficient given the vast composition space of garnet solid solutions. While machine learning has shown promise for predicting emission wavelength, very few models predict thermal stability as a function of composition and site occupancy, representing a major opportunity for future work (Kumar et al., 2025).

Looking forward, three research directions appear particularly promising. First, multi-site synergistic design in high-entropy garnets, where five or more cations occupy a single crystallographic site, provides unprecedented flexibility to tune local structure and rigidity, with early results showing thermal stability far beyond conventional garnets (Li et al., 2026a). Lessons can also be drawn from beyond garnet chemistry: tailoring spin-paired Cr^3+^ dimers in a spinel host has recently afforded giant-tunable and enhanced NIR emission, suggesting that deliberate engineering of activator–activator proximity, and not only its suppression through site isolation, is an additional design degree of freedom for multi-site garnet chemistry (Yang and Jin, 2026). Second, combined defect-site co-engineering that integrates site modification with targeted shallow trap design to produce anti-thermal quenching via thermally activated energy compensation. Third, application-specific site design: for example, luminescence thermometry requires predictable, linear thermal quenching rather than maximum stability, while automotive LEDs require long-term stability against thermal degradation as well as high emission retention at 250 °C (Fang et al., 2022; Zhang et al., 2026). Overall, site engineering remains the most promising route to intrinsically thermally stable garnet phosphors, and continued advances in characterization, computation and material design will enable the next-generation of high-power optoelectronic devices (Zhang et al., 2025a).
